# Relative survival: a useful tool to assess generalisability in longitudinal studies of health in older persons

**DOI:** 10.1186/1742-7622-8-3

**Published:** 2011-02-06

**Authors:** Richard Hockey, Leigh Tooth, Annette Dobson

**Affiliations:** 1 School of Population Health, University of Queensland, Herston QLD 4006, Australia

## Abstract

**Background:**

Generalisability of longitudinal studies is threatened by issues such as choice of sampling frame, representativeness of the initial sample, and attrition. To determine representativeness, cohorts are often compared with the population of interest at baseline on demographic and health characteristics. This study illustrates the use of relative survival as a tool for assessing generalisability of results from a cohort of older people among whom death is a potential threat to generalisability.

**Methods:**

The authors used data from the 1921-26 cohort (n = 12,416, aged 70-75 in 1996) of the Australian Longitudinal Study on Women's Health (ALSWH). Vital status was determined by linkage to the National Death Index, and expected deaths were derived using Australian life tables. Relative survival was estimated using observed survival in the cohort divided by expected survival among women of the same age and State or Territory.

**Results:**

Overall, the ALSWH women showed relative survival 9.5% above the general population. Within States and Territories, the relative survival advantage varied from 6% to 23%. The interval-specific relative survival remained relatively constant over the 12 years (1996-2008) under review, indicating that the survival advantage of the cohort has not diminished over time.

**Conclusion:**

This study demonstrates that relative survival can be a useful measure of generalisability in a longitudinal study of the health of the general population, particularly when participants are older.

## Background

Generalisability (external validity) is the extent to which the results of a study can be applied to other populations. The many threats to the external validity of a study's results include choice of sampling frame, representativeness of the initial sample, and attrition. These issues were discussed in a previous paper [1], and reporting methods were proposed that would enable the reader to assess - at least qualitatively - the generalisability of results from a cohort or longitudinal study. These reporting methods have since been taken further with the publication of the Strengthening the Reporting of Observational Studies in Epidemiology (STROBE) initiative [2].

A common method of assessing generalisability is to compare demographics, health characteristics, and health service variables between a study sample and population of interest at baseline. Over time, this comparison should be repeated to see if biases are changing. This process relies on data from people who enrol and remain in the cohort, enrol and later drop out (using data before withdrawal), were invited but never participated, and those in the population of interest (i.e., all people who might have been selected for inclusion in the study). Repeated assessments of generalisability are particularly appropriate in longitudinal cohort studies where drop out is potentially not a random phenomenon (e.g., related to characteristics of people who cease to participate).

Among cohorts of older people, drop out is frequently due to death. This potential source of bias is different from biases that lead to other types of attrition among participants who are still alive.

Relative survival is the ratio of survival that is observed in the study sample in comparison to that of the population from which it was drawn [3]. This method, which was originally developed to measure survival of cancer patients, has not been used previously to assess bias due to deaths in cohort studies. The main purpose of this paper is to explain and illustrate relative survival as a tool for assessing generalisability of results from a cohort of older people among whom death is a potential threat to generalisability.

We illustrate the method using data from women born in 1921-26 who first participated in the Australian Longitudinal Study on Women's Health (ALSWH) in 1996. We also consider possible reasons for differences in relative survival using data from the ALSWH, data collected from the reference population in the national five yearly census, and periodic national health surveys.

## Materials and methods

### Participants

The ALSWH is a longitudinal study of factors affecting the health and well-being of three national cohorts of women who were born in 1973-78, 1946-51, and 1921-26. The women were selected randomly from the national Medicare health insurance database (which includes all citizens and permanent residents of Australia), with intentional over-sampling of women living in rural and remote areas. In 1996, more than 40 000 women responded to the initial survey; they were reasonably representative of the general population of Australian women in each age group, although compared with data from the 1996 Australian Census there was over-representation of women who were born in Australia, employed and had a university education [4]. More details about the study can be found at http://www.alswh.org.au. Ethical clearance for the study was obtained from the Universities of Newcastle and Queensland.

This paper focuses on the 12 432 women in the 1921-26 cohort who participated in the baseline survey in 1996. Although these women had a nominal age range of 70 to 74 years when the sample was selected, 5% of women were aged 75 years. Due to the small number of participants from the Northern Territory this jurisdiction is not included in the State/Territory comparisons.

### Mortality data

Using personal identifying information provided by the participants, vital status was ascertained by probabilistic linkage to the National Death Index (NDI) for all participants from baseline (1996) to 31 October 2008 [5]. The expected mortality of the study population was ascertained using annual life tables produced by the Australian Bureau of Statistics (ABS) for each State and Territory of Australia [6].

### National Health Survey

The Australian National Health Survey (NHS) is conducted periodically by trained interviewers from the ABS. In addition to demographic information, the survey provides detailed information about the health status of Australians; their use of health services, facilities, and medications; and health-related aspects of their lifestyle. It consists of a representative sample of residents of private and non-private dwellings in all States and Territories, but excludes special dwellings such as hospitals, institutions, and nursing homes.

The 1995 NHS was conducted during the 12-month period of January 1995 to January 1996. It comprised about 23 800 households, representing approximately 57 600 persons [7]. A total of 894 women aged 70 to 74 years (i.e., women born in 1921-25) participated in the 1995 NHS. Using unit record data supplied by the ABS, these women were compared against the ALSWH cohort participants at the first survey for selected characteristics [8].

### Relative survival analysis

Relative survival - the ratio of survival observed in the study sample to the survival to that it should have experienced - can be calculated based on the life table of the population from which it was drawn [3]. In this instance, the study sample was those ALSWH participants born in 1921-26 and the reference population was all Australian women of the same age and State or Territory of residence. It was assumed that the expected mortality experienced by the study sample during a particular period would be the same as mortality in the general population of the same sex, age, and State or Territory of residence from which they are drawn. The Ederer II method was used to calculate interval-specific relative survival [9].

Firstly the population (L) at the start of each interval in each birth cohort and the number of deaths (D) and those lost to follow-up (W) during the interval were determined. From this information, both the population at risk (L') and interval-specific survival (P) were estimated by assuming withdrawals and deaths were evenly distributed over the interval via the formulae:

L'=L−W/2 andP=1−D/L'.

The cumulative survival (CP) for a particular interval (i) was then obtained by the cumulative product of the interval-specific survival terms where the initial cumulative survival (CP(0)) was equal to one, and:

CP(i+1)=CP(i)⋅P(i).

The expected interval-specific and expected cumulative survival (P* and CP*) were calculated similarly, using expected deaths (D*) obtained from appropriate life tables. Finally the interval-specific and cumulative relative survival ratios (R and CR) were calculated as the ratios of the observed and expected interval-specific and cumulative survivals:

$$\begin{array}{l} \text{R}=\text{P}/\text{P}*\text{ and}\\ \text{CR}=\text{CP}/\text{CP}* \end{array}$$

The effect of oversampling women in rural and remote areas in the ALSWH was accounted for via the use of sampling weights, wherein individual weighted deaths both observed and expected were summed to derive the survival estimates.

Separate analyses were carried out for each State and Territory of residence, each category of the Accessibility/Remoteness Index of Australia (ARIA) classification [10], and initial age in years. ARIA categorises areas as 'highly accessible', 'accessible', 'moderately accessible', 'remote' and 'very remote' based on the road distance from the closest service centre. Relative survival was calculated using SAS macros created by Paul Dickman [11].

### Comparison to NHS

Selected demographic, health behaviour, and health status characteristics of the ALSWH sample were compared to those of participants of the 1995 NHS of the same age in order to explore possible reasons for observed differences in survival. These comparisons were presented as percentages and analysed using the χ^2 ^statistic.

### Effects of factors associated with mortality

A proportional hazards model was used in order to assess the effects of initial differences in potential factors associated with mortality between the study sample and the population [12]. The model was of the form:

$$\lambda_{\text{i}}(\text{a},\text{s},{\text{z}}_{\text{i}}(\text{a}))=\lambda^{*}{}_{\text{i}}(\text{a},\text{s})\exp[\beta '{\text{z}}_{\text{i}}(\text{a})]$$

where λ_i_(a, s, z_i_(a)) is the death intensity at age a and State/Territory (s) for the ith individual with covariates z_i_(a) and λ*_i_(a, s) represent the population mortality at age a for an individual of the same sex and State/Territory as the ith individual in the study who is born in the same year as i. The State and Territory specific life tables [6] were used to obtain values for λ*_i_(a, s).

A multiplicative model was used because in the more widely used additive model it is assumed that, at all times and for all values of covariates, the mortality in the study sample is always either higher or lower than that of the general population. This assumption was not justifiable in this context. The effect of oversampling in rural areas was accounted for by including place of residence as it was defined in the original sample (urban, rural and remote) as a covariate in the model. Factors considered in this analysis were based on the results of a previous study of the survival of this cohort [13]. These factors included were: age, marital status, country of birth, State or Territory of residence, Accessibility/Remoteness Index (ARIA), education, smoking status, physical activity, body mass index and self rated health. The proportional hazards model was conducted using the SAS PHREG procedure; a hazard ratio of less than one indicates better relative survival. All analyses were performed using SAS version 9.1 [14].

## Results

### Relative survival

There were 3661 deaths (29.4%) amongst the 12 432 women born in 1921-26 who participated in the baseline survey of the ALSWH. Over the 12-year period of 1996 to 2008, the ALSWH sample had a relative cumulative survival 9.5% (95% confidence interval, 8.3% - 10.7%) greater than their peers in the general Australian population matched for age and State or Territory of residence (Table 1). Interval-specific relative survival remained relatively constant over the whole period, varying between 0.3% and 1.2%.

**Table 1:** Life Table Estimates of Survival of the Australian Longitudinal Study on Women's Health 1921-26 Cohort Relative to Women in the Australian Population Born in the Same Period and Resident in the Same State or Territory.

*Interval* *(years)*	*L*	*D*	*W*	*Effective number at risk*	*Interval-specific observed survival*	*Cumulative observed survival*	*Interval-specific expected survival*	*Cumulative expected survival*	Interval-specific relative survival*	Relative cumulative survival*	*Lower 95% CI*	*Upper 95% CI*
0.0 - 1.0	12424	135	0	12424	0.989	0.989	0.981	0.981	1.008	1.008	1.006	1.010
1.0 - 2.0	12289	181	0	12289	0.985	0.975	0.979	0.960	1.007	1.015	1.012	1.018
2.0 - 3.0	12108	195	0	12108	0.984	0.959	0.977	0.938	1.007	1.022	1.018	1.026
3.0 - 4.0	11912	185	0	11912	0.984	0.944	0.975	0.914	1.010	1.032	1.028	1.037
4.0 - 5.0	11727	237	0	11727	0.980	0.925	0.973	0.890	1.007	1.040	1.034	1.045
5.0 - 6.0	11490	226	0	11490	0.980	0.907	0.971	0.864	1.010	1.050	1.044	1.056
6.0 - 7.0	11264	282	0	11264	0.975	0.884	0.968	0.836	1.007	1.058	1.051	1.064
7.0 - 8.0	10981	359	0	10981	0.967	0.855	0.964	0.806	1.003	1.061	1.054	1.069
8.0 - 9.0	10623	379	0	10623	0.964	0.825	0.961	0.774	1.004	1.066	1.057	1.074
9.0 - 10.0	10244	361	0	10244	0.965	0.795	0.956	0.740	1.009	1.075	1.066	1.085
10.0 - 11.0	9882	422	0	9882	0.957	0.761	0.951	0.704	1.006	1.082	1.071	1.093
11.0 - 12.0	9460	398	707	9106	0.956	0.728	0.945	0.665	1.012	1.095	1.083	1.107

where:

L = Number alive at start of interval;

D = Deaths during interval;

W = Lost to follow-up during each 12 month interval;

Effective number at risk = persons at risk during the interval, accounting for withdrawals;

Interval-specific observed survival = proportion of persons at risk who survive to the end of the interval;

Cumulative observed survival = proportion of persons at risk at the start of the study period who survive to the end of the period, accounting for withdrawals;

Interval-specific expected survival = proportion of persons at risk who are expected to survive to the end of the interval;

Cumulative expected survival = proportion of persons at risk at the start of the study period who are expected to survive to the end of the period, accounting for withdrawals;

Interval-specific relative survival = ratio of observed interval-specific observed survival to expected survival;

Relative cumulative survival = ratio of observed cumulative survival to expected cumulative survival;

* Relative survival ratios greater than one indicate that the study sample has lower total mortality than the population from which they are drawn from while ratios less than one indicate higher mortality;

ALSWH participants had significantly better survival than the general population in all jurisdictions, with their relative cumulative survival advantage ranging from 6% in South Australia to 23% in the Australian Capital Territory (Table 2). Their relative cumulative survival was consistently higher than for the general population across all ARIA groups, although in the remote/very remote areas the difference was not statistically significant (Table 2). The relative survival advantage of ALSWH participants increased with initial age: among those assessed in 1996, a 6% advantage among women aged 70 approached 22% among women aged 75 (Table 2).

**Table 2:** Life Table Estimates of Relative Cumulative Survival Over the Period 1996-2008 Among Participants in the Australian Longitudinal Study on Women's Health 1921-26 Cohort by State or Territory of Residence, Accessibility/Remoteness Index (ARIA), and Age at Baseline (1996).

	Relative Cumulative Survival	95% Confidence Limits	
		Lower	Upper
**State or Territory of Residence**			
Australian Capital Territory	1.232	1.113	1.321
New South Wales	1.108	1.087	1.127
Queensland	1.085	1.054	1.113
South Australia	1.063	1.024	1.099
Tasmania	1.106	1.024	1.179
Victoria	1.086	1.063	1.109
Western Australia	1.109	1.068	1.146
**Accessibility/Remoteness Index (ARIA)**			
Highly Accessible	1.104	1.091	1.116
Accessible	1.042	1.004	1.079
Moderately Accessible	1.064	0.997	1.124
Remote, Very Remote	1.068	0.939	1.173
**Age at Survey 1 (years)**			
70	1.064	1.039	1.087
71	1.060	1.036	1.083
72	1.094	1.068	1.119
73	1.109	1.079	1.138
74	1.129	1.095	1.161
75	1.215	1.145	1.280

### Comparison of ALSWH cohort to NHS sample at baseline

The comparison of the ALSWH 1921-26 cohort at baseline (1996) with the 1995 NHS for selected socio-demographic characteristics and health related behaviours is shown in Table 3. The ALSWH cohort was less likely than their NHS counterparts to be widowed, more likely to be married, and more likely to have a tertiary education. There were more women born in 'Other English speaking background (ESB)'countries in the ALSWH cohort and less born in Europe. Among health related behaviours, ALSWH participants were less likely to be current smokers, report fair or poor self-rated health, and report that their health limited their ability to exercise (walk 100 metres).

**Table 3:** Comparison of Selected Characteristics Between the Australian Longitudinal Study on Women's Health 1921-26 Cohort and the 1995 National Health Survey.

	ALSWH N = 12423	NHS N = 894	
Item	%	%	*P *Value
***Smoking Status***			
*Never-smoker*	62.1	64.6	<0.0001
*Ex-smoker*	30.4	24.3	
*Current smoker*	7.6	11.0	
***Marital Status***			
*Partnered*	55.6	49.6	<0.0001
*Separated/Divorced*	6.3	5.6	
*Widowed*	34.8	42.4	
*Never married*	3.2	2.4	
***Country of Birth***			
*Australian born*	73.5	74.2	0.05
*Other English Speaking*	13.6	10.9	
*Europe*	10.1	12.0	
*Asia*	1.8	1.4	
*Other*	1.0	1.6	
***Highest Educational Qualification***			
*No Higher Qualification*	84.0	79.3	<0.0001
*Trade/Apprentice Certificate/Diploma*	11.7	16.7	
*University*	4.2	2.7	
*Inadequately described*		1.2	
***Body Mass Index (BMI) Group***			
*Underweight, BMI < 18.5*	3.2	4.2	0.07
*Healthy weight, 18.5 ≤ BMI < 25*	50.4	52.1	
*Overweight, 25 ≤ BMI < 30*	33.1	29.1	
*Obese, 30 ≤ BMI*	13.2	14.5	
***Self Rated Health***			
*Excellent*	6.4	7.6	<0.0001
*Very good*	26.2	23.2	
*Good*	39.4	34.4	
*Fair*	23.6	24.3	
*Poor*	4.3	10.5	
***Does Your Health Limit You in Walking 100 m***			
*Limited a lot*	7.1	11.3	<0.0001
*Limited a little*	15.4	19.7	
*Not limited*	77.4	68.9	
***State or Territory of Residence***			
*New South Wales*	34.9	35.8	0.969
*Australian Capital Territory*	1.1	0.8	
*Queensland*	16.3	16.6	
*South Australia*	10.2	10.0	
*Tasmania*	2.8	2.5	
*Victoria*	26.0	25.9	
*Western Australia*	8.5	8.5	

### Effects of factors on mortality (relative survival)

The results of the multiplicative relative survival model are shown in Table 4. Relative survival was significantly associated with initial age, country of birth, State or Territory of residence, marital status, body mass index (BMI), smoking status, physical activity, and self rated health. For example, age at baseline was positively associated with better relative survival, implying that - even after adjusting for major risk factors - older members of the cohort were healthier than their younger counterparts in the general population.

**Table 4:** Multivariate Analysis of Relative Survival of the Australian Longitudinal Study on Women's Health 1921-26 Cohort, 1996-2008.

	Hazard Ratio*	*95% Confidence Limits*	
*Item*		*Lower*	*Upper*
***Smoking Status***			
*Never-smoker (Ref)*	1.00		
*Ex-smoker*	1.27	1.16	1.38
*Current smoker*	1.84	1.61	2.10
***Marital Status***			
*Partnered (Ref)*	1.00		
*Separated/Divorced*	1.09	0.92	1.30
*Widowed*	1.10	1.01	1.20
*Never married*	1.36	1.08	1.72
***Country of Birth***			
*Australian born (Ref)*	1.00		
*Other English Speaking*	1.02	0.90	1.15
*Europe*	0.85	0.73	1.00
*Asia*	0.64	0.42	0.97
*Other*	0.38	0.20	0.74
***Highest Educational Qualification***			
*No Higher Qualification (Ref)*	1.00		
*Trade/Apprentice Certificate/Diploma*	0.97	0.85	1.11
*University*	0.86	0.68	1.08
***Body Mass Index (BMI) Group***			
*Underweight, BMI < 18.5*	1.66	1.39	1.97
*Healthy weight, 18.5 ≤ BMI < 25 (Ref)*	1.00		
*Overweight, 25 ≤ BMI < 30*	0.83	0.76	0.91
*Obese, 30 ≤ BMI*	0.96	0.85	1.08
***Self Rated Health***			
*Excellent (Ref)*	1.00		
*Very good*	1.10	0.88	1.37
*Good*	1.42	1.14	1.75
*Fair*	2.42	1.95	3.00
*Poor*	4.86	3.80	6.22
***Physical Activity***			
*None*	1.60	1.44	1.78
*Low*	1.04	0.93	1.17
*Moderate (Ref)*	1.00		
*High*	1.04	0.89	1.20
***State or Territory of Residence***			
*New South Wales (Ref)*	1.00		
*Australian Capital Territory*	0.88	0.51	1.53
*Queensland*	1.09	0.97	1.22
*South Australia*	1.09	0.94	1.26
*Tasmania*	1.06	0.86	1.30
*Victoria*	1.13	1.01	1.25
*Western Australia*	1.07	0.90	1.26
***Accessibility/Remoteness Index (ARIA)***			
*1. Highly Accessible (Ref)*	1.00		
*2. Accessible*	1.10	0.99	1.21
*3. Moderately Accessible*	1.18	1.00	1.39
*4. Remote, Very Remote*	1.18	0.85	1.64
***Age at Survey 1 (years)***			
*70 (Ref)*	1.00		
*71*	1.07	0.93	1.23
*72*	0.98	0.85	1.12
*73*	0.92	0.80	1.06
*74*	0.91	0.79	1.05
*75*	0.73	0.59	0.90

* Hazard ratios greater than one indicates higher mortality relative to the reference category while ratios less than one indicate lower mortality;

## Discussion

In the 12 years (1996-2008) under consideration, the ALSWH 1921-26 cohort had significantly better survival than the general population. This better relative survival was consistent across all jurisdictions for the duration of the study. There was also no indication that the survival of the sample converged to that of the general population over time. This result was unexpected because most sample groups tend to become more like the general population over time (or, even if they differ at baseline, the effect of that initial difference becomes less important). Other longitudinal studies have found the mortality of sampled respondents and non-respondents converging over time [15,16].

That the ALSWH 1921-26 cohort had significantly better survival was expected, as participants were self selected from an initial sample of randomly selected Australian women. From an initial sample of 39 000 women selected from the Medicare database, 12 432 responded [17]. It would be expected that older women - in particular those with better health ('the healthy volunteer') or who were more interested in their health - would be more likely to participate in such a survey. Similar effects have been observed in other longitudinal studies [15,16,18].

Consistent with previous analysis comparing the ALSWH cohort to census data [19], systematic differences were observed between the ALSWH 1921-26 cohort and the participants of the 1995 National Health Survey. A previously published study of survival among the 1921-26 cohort showed that self rated health was a strong predictor of long term survival [13]. Other variables found to be associated with survival in this current study (e.g., marital status, country of birth, smoking, physical activity and BMI) were also associated in the previous study. This study found significant differences between the ALSWH cohort and the NHS participants with respect to several variables, namely: self rated health, smoking status, marital status, and country of birth. Given the magnitude and direction of these differences, these factors could explain a major portion of the observed survival advantage in the ALSWH cohort. For example, if the distribution of self rated health observed in the NHS was applied to the ALSWH sample then the cumulative mortality of the ALSWH cohort as a whole would be increased by 10%, thereby reducing the survival advantage observed in the ALSWH cohort by about a half.

### Limitations

An alternative possible reason for the observed difference in survival between the ASLWH sample and NHS participants could be incomplete ascertainment of deaths in the study population. Death information was obtained from both linkage to the NDI as well as notification by family or carers of participants. A previous paper examining linkage of the study population to the NDI in 1998 showed that such a linkage identified 95% of the deaths [5], however this has not been reassessed since that time and it is possible that this capture method has worsened over the last decade. Other studies of the accuracy of the NDI found the false negative rate ranging from 3% to 11% (compared to 5% in the ALSWH study) [20-22]. If the false negative rate is as high as 10%, this could account for about half the difference observed in this study. However, such a high false negative rate is considered unlikely because NDI linkage is supplemented by other information, particularly data that is obtained at the time of the triennial ALSWH surveys. Still, the systematic difference in survival observed over the study period suggests some under ascertainment of deaths may have occurred.

Another limitation to the use of relative survival as a tool for assessing generalisability is the categorisation of available life tables. For Australia, life tables are available by age, sex and State/Territory of residence only; it would be useful if they were available for other factors such as smoking status. Indeed, if other population data on survival were available with stratification by other variables (e.g. from other cohort studies), then the relative survival approach with weighting by strata would be feasible.

## Conclusion

This study has shown that relative survival can be a useful and relatively easily obtained measure of generalisability (external validity) in a longitudinal study of the health of a population-based sample, particularly when participants are of advanced years. The advantage of this method is that, through a single measure, it can indicate the degree to which a study sample corresponds to the general population with respect to health status. Along with the other comparisons of the study population to census and survey data, this measure provides information relevant to the reporting requirements of the STROBE statement [2]. In the case of the ALSWH cohort studied here, it seems likely that most of the difference in their survival over that of the general population is attributable to the better health of the sample at baseline.

### Implications

It is essential that any future analysis of this cohort considers the results of this investigation, but if and how any adjustments to design must occur will depend on the objectives of future work. If the analysis involves examining the associations of various factors with some outcome, then it may be sufficient to control for the factors that were found to be associated with improved survival. On the other hand, if population estimates are required then it would be necessary to employ some type of weighting scheme involving these factors, as well as the weights that account for the deliberate oversampling in rural areas. For example, if one was estimating the population prevalence of diabetes in older women then it may be necessary to use weights for area of residence and perhaps other factors such as self rated health.

## Competing interests

The authors declared no conflict of interest, and no funding source was involved in the creation of this manuscript.

## Authors' contributions

RH conceptualized the study, conducted the analysis, and prepared the manuscript; LT and AD assisted with the initial design, methods used in the analysis, and drafting of the manuscript. All authors have read and approved the final manuscript.
